# Neonatal Hyperglycemia Related to Parenteral Nutrition Affects Long-Term Neurodevelopment in Preterm Newborn: A Prospective Cohort Study

**DOI:** 10.3390/nu13061930

**Published:** 2021-06-04

**Authors:** Giovanni Boscarino, Maria Giulia Conti, Corinna Gasparini, Elisa Onestà, Francesca Faccioli, Lucia Dito, Daniela Regoli, Alberto Spalice, Pasquale Parisi, Gianluca Terrin

**Affiliations:** 1 Department of Maternal and Child Health, Policlinico Umberto I Hospital, Sapienza University of Rome, 00161 Rome, Italy; giovanni.boscarino@yahoo.com (G.B.); mariagiulia.conti@uniroma1.it (M.G.C.); corinna.gasparini@uniroma1.it (C.G.); elisa.onesta@gmail.com (E.O.); francesca.faccioli@hotmail.it (F.F.); lucia.dito@yahoo.it (L.D.); dani.regoli@virgilio.it (D.R.); alberto.spalice@uniroma1.it (A.S.); 2 Department of Molecular Medicine, Sapienza University of Rome, 00185 Rome, Italy; 3 NESMOS Department, Faculty of Medicine & Psychology, c/o Sant’ Andrea Hospital, Sapienza University, 00189 Rome, Italy; pasquale.parisi@uniroma1.it

**Keywords:** mortality, neurodevelopment, Bayley Scales of Infants Development, VLBW, metabolic complication, maternal age

## Abstract

(1) Background: Recent evidence reported a reduced tolerance of macronutrient parenteral intakes in subjects in critically ill conditions. We designed a prospective cohort study to evaluate the effects of hyperglycemia (HG) related to parenteral nutrition (PN) on neurodevelopment (NDV) in survived preterm newborns. (2) Methods: Enrolled newborns with gestational age < 32 weeks or birth weight < 1500 g, were divided in two cohorts: (A) exposed to moderate or severe HG (glucose blood level > 180 mg/dL) in the first week of life; (B) not exposed to HG. We considered as the primary outcome the rate of preterm newborns survived without NDV delay at 24 months of life, evaluated with Bayley Scales of Infants Development III edition. (3) Results: We analyzed 108 (A 32 vs. B 76) at 24 months of life. Newborns in cohort A showed a higher rate of cognitive and motor delay (A 44% vs. B 22 %, *p* = 0.024; A 38% vs. B 8%, *p* < 0.001). When adjusting for background characteristics, HG remained a risk factor for motor delay. (4) Conclusions: High nutritional intakes through PN soon after birth increase the risk of HG. The consequences of this severe metabolic complication affect long-term NDV and survival in preterm newborns.

## 1. Introduction

It has been reported that, in developed countries, the survival rate of VLBW newborns is about 90% [1]. Up to 60–70% of this population develop extrauterine growth retardation (EUGR) [2]. To limit EUGR in preterm newborns, current guidelines recommend to administer high nutritional intakes in parenteral nutrition (PN) soon after birth [3]. It has been reported that EUGR affects long-term neurodevelopment (NDV) [4]. However, long-term efficacy of this nutritional approach in order to reduce EUGR, and thus to improve NDV in children born preterm, is still debated [5,6,7,8]. We have previously demonstrated that energy-enhanced PN early in life does not improve post-natal growth and results in lower motor and socioemotional competence performance at 24 months of corrected age in preterm babies independently to long-term growth [5,6].

Recent evidence reported a reduced tolerance of macronutrient parenteral intakes in adults and children in critically ill conditions [9,10,11,12]. A randomized control trial including critically ill neonates at term demonstrated that withholding PN for 1 week was clinically superior to starting PN soon after birth for short-term outcomes [12].

Hyperglycemia (HG), considered a marker of tolerance of energy intake given by PN, has been associated with an increased rate of mortality in preterm newborns receiving enhanced PN in comparison with standard PN in the first week of life [13]. The incidence of HG in preterm babies ranges from 15% to 30%, in relation to the definition of the threshold [14]. The occurrence of HG in early life has also been related to an impaired white matter development revealed by magnetic resonance imaging (MRI) and to a worsening long-term NDV in recent observational and case-control study [15,16].

In light of these considerations, we supposed that the occurrence of HG, related to PN, could influence the rate of long-term NDV delay in children born preterm. To demonstrate our hypothesis, we designed a prospective cohort study to evaluate the influence of HG related to early-enhanced PN on NDV in survived preterm babies.

## 2. Materials and Methods

### 2.1. Study Design and Population

We considered eligible all newborns with gestational age (GA) < 32 weeks or body birth weight (BW) < 1500 g, consecutively admitted from January 2015 to December 2019 to the neonatal intensive care unit (NICU) of Policlinico Umberto I Hospital in Rome, requiring PN in the first week of life. Newborns with congenital diseases, inborn errors of metabolism, congenital infections, and hospital discharge or death within 72 h of life in terminal condition were excluded [17,18,19,20].

Blood glucose levels were monitored by validated micro-method, four to eight times a day from the first days of life (DOL) and less frequently when the clinical conditions were stabilized, from capillary blood and analyzed by point of care device Accu-Chek Inform II glucometer (Roche, Indianapolis, IN, USA) [21]. The HG was defined as two consecutive blood glucose levels greater than 180 mg/dL, at least 3 h apart, and was categorized as moderate (181–239 mg/dL) or severe (>240 mg/dL). Enrolled newborns were divided into two Cohorts: (A) newborns exposed to moderate or severe HG in the first week of life; (B) neonates not exposed to HG.

### 2.2. Outcome

We considered as the primary outcome the rate of preterm newborns survived without NDV delay at 24 months of life. We also evaluated the occurrence rate of major prematurity-related morbidity, length of hospital stay, and EUGR.

### 2.3. Data Collection

Medical staff, blinded to the study aims, evaluated the eligibility criteria. They were in charge of the babies and monitored the blood glucose levels. Researchers not involved in the NICU clinical practice and unaware of the study aims recorded information in a specific data form for statistical analysis, which was performed by a blinded statistician. A third-party observer, not involved in clinical practice, collected data on NDV.

Prenatal, perinatal, and postnatal data were prospectively recorded, as previously described [6]. We daily collected nutritional intake on PN, enteral nutrition (EN), and feeding tolerance.

As previously described [5], we defined morbidity the presence of at least one of the major prematurity-related complications, including necrotizing enterocolitis (NEC) Bell-Stage ≥ II, periventricular leukomalacia (PVL), late-onset culture proven sepsis, retinopathy of prematurity (ROP), and bronchopulmonary dysplasia (BPD). Diagnosis of prematurity-related morbidities was performed according to standard criteria [22,23,24,25,26]. We considered EUGR, with longitudinal definition, as the loss of 1 standard deviation (SD) from birth to 36 weeks of PMA [27].

Children were assessed at 24 months of life with the cognitive, language, and motor scales of the BSID III by a trained psychologist blinded to the study aims [28]. We considered a standardized score <1 standard deviation (SD) from the test mean to define NDV delay [29,30].

### 2.4. Hyperglycemia and Blood Glucose Management

The definition of moderate HG corresponds to our unit’s operational threshold to reduce the glucose infusion rate. While we initiated insulin infusion in newborns presenting sever HG. Moderate HG was treated by reducing the intravenous glucose concentration by 1–2 mg/kg/min. If this did not reduce the blood sugar within 4 h, the dextrose was again decreased until a minimum of 5 mg/kg/min. Insulin infusion was set at 0.1 U/kg/h and titrated (every 4 h) to maintain blood sugar under 180 mg/dL. The minimum dose of insulin use was 0.001 U/kg/h, and the maximum was 1.0 U/kg/h.

### 2.5. Nutritional Protocol

The amount of macro and micronutrients administered through PN was in line with the actual EPSGHAN guidelines for PN during the study period [3]. Until full enteral feeding (FEF, 120 kcal/Kg/day) was achieved, we administered PN via a central venous access device to maintain adequate fluids, electrolytes, and nutrient intakes, soon after birth [31,32]. Mother’s own milk was administered as soon as possible after birth and donor milk was not available during the study period [33]. Preterm formula was administered when human milk was not available or sufficient. Minimal enteral feeding (MEF), started at 10–20 mL/kg/day, was increased by 20–30 mL/kg/day if enteral nutrition was tolerated [34,35]. We defined total PN (TPN) when PN represents more than 70% of energy of total nutrition (enteral and parenteral) in the first 7 DOL.

### 2.6. Statistics

Statistical analysis was performed using Statistical Package for Social Science software (SPSS Inc., Chicago, IL, USA), version 25.0. We checked for normality using the Shapiro–Wilk test. The mean and 95% confidence interval (CI) summarized continuous variables, while the number and percentage described categories variables. We used the χ^2^ test for categorical variable and *t*-test or Mann–Whitney for paired and unpaired variables. To evaluate the influence of PN independently to enteral feeding, we selected, in a sensitivity analysis, newborns nourished with TPN in the first 7 DOL.

We performed a binary regression analysis to evaluate the influence of PDA, invasive mechanical ventilation support, clinical risk index for babies (CRIB) II score, maternal age ≥ 35 years old, antenatal corticosteroids, and HG on the rate of death, EUGR, and morbidity. A binary regression analysis was also performed to evaluate the influence of covariates statistically significant in univariate model (PDA, CRIB II score, two doses of antenatal corticosteroids administration, start of EN before to 72 h of life, maternal age ≥ 35 years, ventilation support, and energy intake by PN on the first week of life ≥432 kcal/kg/week) on the rate of HG. The cut-off of 432 kcal/kg/week was the 50° percentile of the energy intake of the study population with HG. We performed two models of logistic regression analysis considering the NDV delay in each domain of BSID-III as dependent variable and maternal age ≥ 35 years, EUGR, HG, IVH, sex, ethnicity, and GA ≤29 weeks of PMA (model I) or ELBW (model II) as confounding variables. The level of significance for all statistical tests was two-sided (*p* < 0.05).

## 3. Results

**Figure 1:**
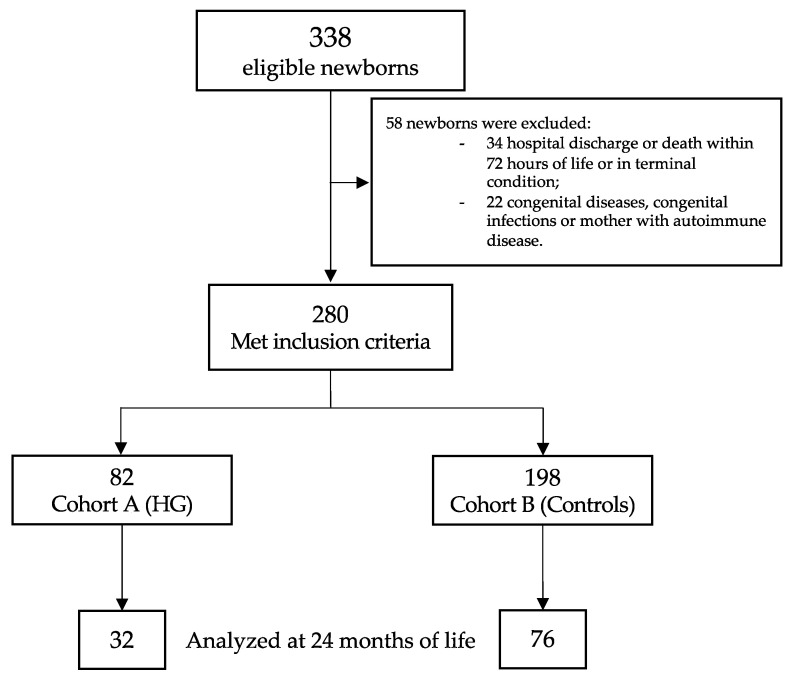
Flow chart. Figure legend: HG (hyperglycemia).

Of the 338 eligible newborns, 280 met the inclusion criteria (Figure 1). Table 1 showed the baseline clinical characteristics of the study population. Newborns with HG showed a higher rate of mother with more than 35 years of life, lower GA and BW, higher CRIB II score, higher rate of mechanical ventilation support (for both invasive and non-invasive), and higher rate of PDA (Table 1).

Survival was higher in cohort B compared with the newborns in cohort A (99.0% vs. 86.6%, OR 15.183, *p* < 0.001). Morbidity was higher in cohort A compared with cohort B, as shown in Table 2. In particular, the rate of IVH, sepsis, ROP, and BPD was higher in cohort A (Table 2). Length of hospital stay was longer in cohort A compared with cohort B (80 days 95 % CI 68 to 92 days vs. 54 days 95 % CI 51 to 57 days, *p* < 0.001). We did not find differences in the rate of EUGR between the two study cohorts (A 59.8% vs. B 60.6%, OR 0.965, *p* = 0.895).

**Figure 2:**
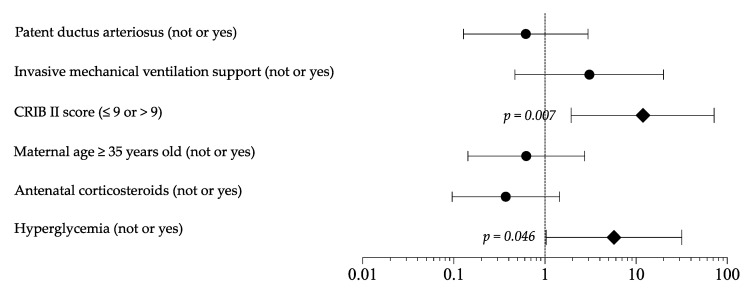
Logistic regression analysis to evaluate the influence of covariates on mortality rate. Figure legend: CRIB (clinical risk index for babies).

As shown in Tables S1 and S2, the sensitivity analysis, including only newborns receiving TPN in the first 7 DOL, confirmed the differences in baseline clinical characteristic, survival (A 98.7% vs. B 86.8%, OR 11.898, *p* = 0.004), morbidity, length of hospital stay (A 82 days 95 % CI 68 to 96 days vs. B 67 days 95 % CI 62 to 73 days, *p* = 0.035), and EUGR (A 60.3% vs. B 65.8%, OR 0.788, *p* = 0.488). Logistic regression analysis showed that HG and CRIB score >9 represented independent risk factor for mortality (Figure 2). In the multivariate model, HG appeared to not be related to EUGR and morbidity rate.

**Figure 3:**
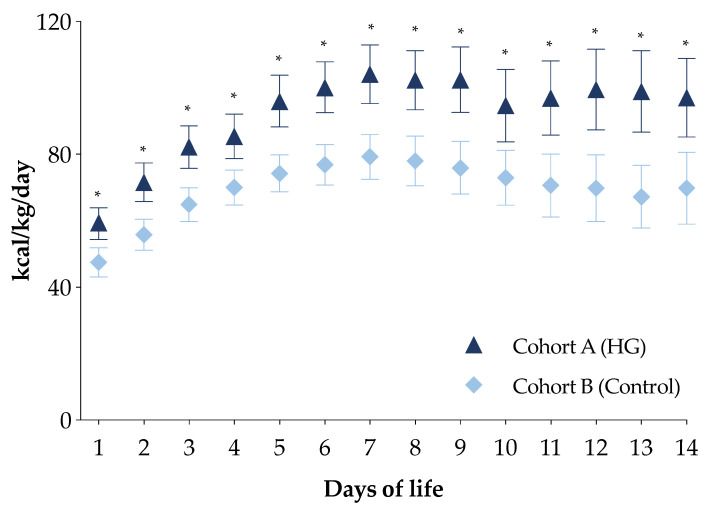
Daily energy intake of the first two weeks of life of the two study cohorts in total parenteral nutrition. Figure legend: HG (Hyperglycemia); * *p*-value < 0.05.

The PN energy intakes in the first week of life of the newborns in cohort A were higher compared with the newborns in cohort B (Figure S1). In particular, newborns in cohort A received a higher intake of all macronutrients compared with those in cohort B (Figure S1). Moreover, daily energy intake by PN in the first two weeks of life for this subpopulation was higher in newborns of cohort A compared with those of cohort B (Figure 3).

**Figure 4:**
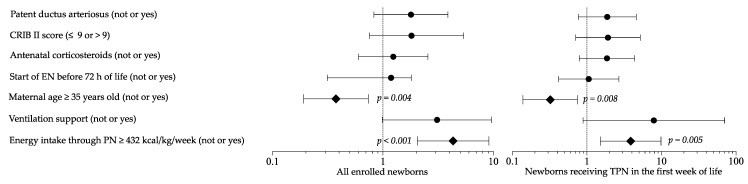
Multivariate analysis to evaluate the influence of covariates on the occurrence of hyperglycemia. Figure legend: CRIB (clinical risk index for babies); EN (enteral nutrition); PN (parenteral nutrition); TPN (total parenteral nutrition).

Logistic regression analysis showed that maternal age and high energy intake in PN were risk factors for HG in a multivariate model (Figure 4). Sensitivity multivariate analysis including newborns in TPN confirmed these results (Figure 4).

**Figure 5:**
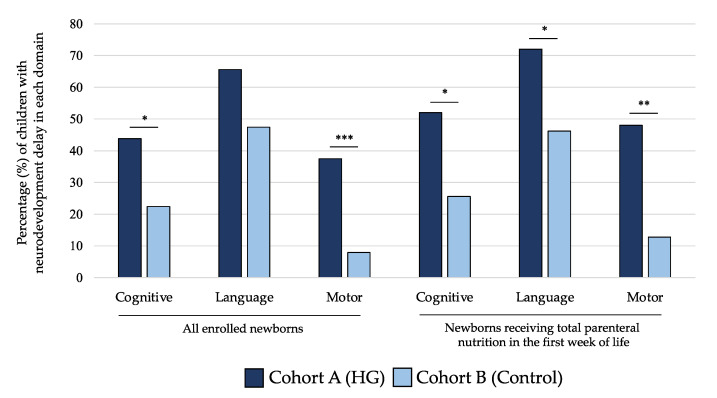
Percentage of children with neurodevelopment delay in each domain of Bayley Scale of Infant Development at 24 months of life of the two study cohorts. Figure legend: HG (hyperglycemia); * *p*-value < 0.05; ** *p*-value < 0.01; *** *p*-value < 0.001.

As shown in Figure 5, the rate of NDV delay at 24 months of life for cognitive and motor scales of BSID-III was significantly higher in children of cohort A compared with those of cohort B. When we analyzed only subjects receiving TPN in the first 7 DOL, we observed that newborns in cohort A showed higher rate of NDV delay in all domains of BSID-III (Figure 5). In Table 3, we showed that mean values of performances in cognitive, language, and motor domains were lower in subjects included in cohort A compared with cohort B at 24 months for all populations of newborns analyzed in the sensitivity analysis.

**Figure 6:**
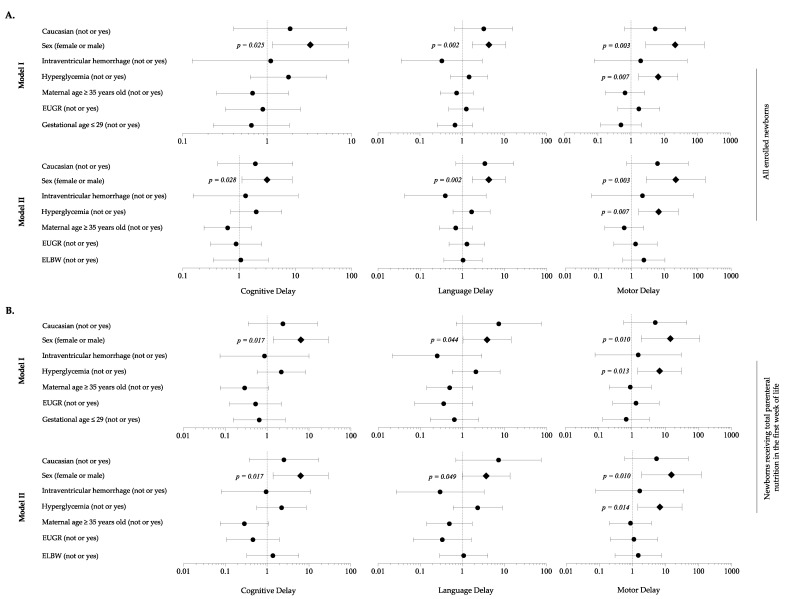
Multivariate analysis to evaluate the influence of covariates on the occurrence of neurodevelopment delay in each domain of Bayley Scales of Infants Development at 24 months of life. (**A**) All enrolled newborns; (**B**) Newborns receiving total parenteral nutrition in the first week of life. Figure legend: EUGR (extrauterine growth retardation); ELBW (extremely low birth weight infants).

When adjusting for background characteristics, analysis revealed that HG was a risk factor for motor delay, in association with male sex (Figure 6A). Sensitivity analysis considering newborns in TPN during the first 7 DOL confirmed these findings (Figure 6B).

## 4. Discussion

Our findings reveled that HG has a significant impact on survival in a population of children born preterm in critically ill condition. We further demonstrated that HG depended on energy intake given by PN in the first 7 DOL and on maternal age. Finally, we showed that HG and male sex are risk factors for neurological impairment at 24 months of life.

The effects of HG on long-term NDV are still debated and there are similar observations in other jurisdictions. In a prospective study, Gonzalez Villamizar et al. aimed at evaluating the effects of HG on body composition and NDV, and how early nutrition and illness modify these relationships in infants born preterm [36]. The authors demonstrated that, in infants born before 32 weeks of PMA, more than 5 days of HG were associated with decreased lean mass at 4 months’ PMA and poorer NDV outcome at 12 months’ PMA. These observations may be owing to an overall decrease in the nutritional intakes in the first week, as a consequence of the reduced glucose infusion rate needed for the management of HG. Some limitations affect the results of the aforementioned study. First, the number of glucose measurements recorded for each enrolled patient varied according to the discretion of the clinician, which could underestimate the true glycemic state of each patient. Second, the high rate of drop-out at follow-up may make the results less representative of the original sample of patients recruited. Van der Lung et al., in a retrospective study, showed that HG was associated with a worsening of NDV evaluated by a neurological examination at 2 years of life [16]. However, these results were not corrected for confounding variables. In addition, an observer-bias cannot be excluded because several physicians did the follow-up consults. The composition of the unexposed control group was randomly chosen and purely based on the in advance defined matching criteria. Thus, an unintentional selection bias cannot be excluded. Finally, NDV was assessed by neurological examination and not by a standardized test as BSID. Ramel et al. demonstrated that neonatal HG was not associated with lower scores on the Bayley scales evaluated at 12 and 24 months of life [37]. However, the author did not report nutritional intake and did not perform a multivariate analysis, including variables that may have an important role in long-term NDV. In addition, this study is limited by its retrospective design. In a recent study including critically ill term newborns, Verlinden et al. evaluated the effects of two nutritional strategies: the early start of PN (early-PN) versus withholding it for one week (late-PN) [11]. Two years later, long-term development of neurocognitive, behavioral, and emotional functions were improved in children in the late-PN group. These authors suggest a de-implementation of nutritional strategies characterized by high energy and protein intake in critically ill children of all ages, but especially for critically ill children aged between 29 days and 11 months at the time of exposure to PN [11]. In our study, NDV was assessed at 24 months of life by a single phycologist and through a standardized test. We then confirmed the effects of HG on NDV, correcting for confounding variables.

We observed that NDV impairment depends also on male sex other than HG. This relatively poor NDV outcome in preterm males may reflect increased prevalence of neonatal brain abnormalities rather than an independent sex-specific response to HG. Previous evidence indicates that HG is more common in preterm females [38] and that higher incidence of abnormal NDV in preterm males relates to greater incidence and severity of brain abnormalities [39].

It has been demonstrated in observational studies that HG is a risk factor for death in critical premature infants [15,40,41,42]. In addition, Stensvold et al., in a recent prospective cohort study, evaluated the influence of HG on mortality rate [13]. The authors demonstrated that infants enrolled in the cohort with an enhanced PN protocol have a higher risk of mortality (OR 2.64; 95% CI, 1.39–4.98), after an implementation of nutritional protocol [13]. In the multivariate models, these authors included HG and PN energy intake as covariates. However, on the basis of our results, occurrence of HG may be influenced by PN energy intake. In addition, the authors of this study did not evaluate the factors influencing the occurrence of HG [13].

In vitro, Temming et al. demonstrated an increased proinflammatory cytokine response in the blood of preterm and term neonates with HG; thus, HG induces oxidative stress and inflammatory reactions that support the hypothesis of a direct relationship between neonatal high blood glucose levels and adverse outcome, in contrast to the idea that detrimental effects of HG in the human neonate are merely a reflection of critical illness [43]. In addition, an animal model study demonstrated that high glucose levels activate the caspase with a consequent reactive oxygen species (ROS) production, responsible for triggering an apoptotic process in the brain [44]. This process is significant in the hippocampus, in which increased levels of caspase -3, caspase -8, and caspase -9 were found [44]. Tayman et al. in animal models conclude that the severity of HG causes cell death in the developing brain, decreases brain density, and affects the development of brain tissues in the neonatal period [44]. In addition, we observed that maternal age over 35 years represents a protective factor for HG. It has been demonstrated that, in twin pregnancy, older maternal age is associated with indices of insulin resistance [45]. Despite that little is known in singleton, it is possible to speculate that maternal insulin resistance may improve tolerance of glucose in fetus, and thus in preterm newborns with similar GA. However, gestational diabetes was not related to HG in our study. This hypothesis should be confirmed in a specifically designed study.

Despite being interesting, our results should be interpreted considering several limitations. Our findings may be related to the effects of chance (random error), bias, or confounding factors. We verified that the effects on NDV of HG persisted even after correcting for confounding variables. Despite everything, unknown confounding variables or ones not considered in our statistical analysis may have influenced the study results. Indeed, neurological development is complex, with endogenous and exogenous factors at play [46,47]. We evaluated in a binary regression analysis the possible factors that could influence the occurrence of HG. A possible confounding factor is the early (<8 DOL) post-natal administration of corticosteroids [48]. We did not include this factor in our model because we considered HG in the first 7 DOL and we had no subjects treated with corticosteroids in this time frame. Moreover, this is not a RCT. Individualized nutritional corrections are the milestone of our policy on PN, in order to avoid deleterious consequences of complications related to the administration of intravenous macronutrients [31]. Despite being a potential information bias, we have preferred that physicians taking care of babies were aware of the composition of PN, in order to make immediate corrections in the case of complications. In addition, the risk of lack of equipoise within neonatologists caring for preterm infants could be very high. Hence, it is not easy to design an RCT in newborns in critically ill conditions. On the other hand, the severity of clinical conditions may increase the use of PN. To exclude confounding effects of this aspect, we confirmed our results in a sensitivity multivariate analysis including only subjects who were fed mainly by PN in the first 7 DOL. A reduction of energy intake in PN secondary to HG could be associated with a reduced energy intake in PN in newborns with HG compared with controls. However, the PN intake of the first 14 DOL remained higher in cohort A compared with cohort B, not influencing the rate of EUGR between the two cohorts of the study. To limit selection bias, neonatologists evaluating eligibility used objective inclusion criteria (such as GA and BW), unaware of the study aims. In addition, researchers not involved in clinical practice and eligibility assessment and who were unaware of the cohort assignment collected the data for the statistical analysis. A protocol for the collection, measurement, and interpretation of data was discussed and defined before starting the study. It has been demonstrated that BSID-III tends to underestimate neurodevelopmental delay compared with other scales [49]. However, measurement of NDV using different scales, exploring further domains other than those evaluated by BSID, could overcame this bias. A blinded third part observer collected data on the primary outcome of the study and a blinded statistician performed the analysis. Despite no changes in the policies care during the study period and similar baseline characteristics of the two cohorts, it is not possible to exclude that unknown differences in the clinical practice or changes in the medical staff composition may have influenced the results.

## 5. Conclusions

High nutritional intakes through PN in the first 7 DOL increase the risk of HG. The consequences of this severe metabolic complication affect survival and NDV at 24 months of life. Our data suggest a reduction of energy intake in PN in the first week of life. Further randomized controlled trials are urgently needed to confirm the negative role of enhanced PN soon after birth, for brief-term metabolic and, consequently, long-term neurological outcomes of babies born before 32 weeks of PMA or VLBW. As critically ill children aged 29 days to 11 months at time of exposure are most vulnerable to developmental harm evoked by early-PN [11], an early-enhanced PN could also be deleterious for long-term NDV of preterm babies.

## Figures and Tables

**Table 1:** Baseline clinical characteristics of the study population.

	Total *n =* 280	Cohort A (HG) *n =* 82	Cohort B (Control) *n =* 198	OR (95% CI)	*p* Value #
Maternal age, years old	34 (33 to 35)	34 (33 to 35)	35 (34 to 35)	-	0.364
Maternal age ≥ 35 years old, No. (%)	132 (47.1)	61 (74.4)	71 (35.9)	0.422 (0.243–0.731)	0.002
Gestational age, weeks	29 (29 to 30)	27 (27 to 28)	30 (29 to 30)	-	<0.001
Gestational age ≤ 29 weeks, No. (%)	148 (52.9)	21 (25.6)	127 (64.1)	0.192 (0.108–0.342)	<0.001
Birth weight, g	1248 (1207 to 1291)	1010 (936 to 1085)	1347 (1303 to 1391)	-	<0.001
Birth weight ≤ 1000 g, No. (%)	72 (25.7)	44 (53.7)	28 (14.1)	7.030 (3.897–12.683)	<0.001
Male sex, No. (%)	151 (53.9)	43 (52.4)	108 (54.5)	0.919 (0.549–1.539)	0.748
Caucasian, No. (%)	231 (82.5)	63 (76.8)	168 (84.8)	1.689 (0.887–3.214)	0.108
Cesarean section, No. (%)	243 (86.8)	70 (85.4)	173 (87.4)	0.843 (0.401–1.771)	0.652
Antenatal corticosteroids ^a^, No. (%)	195 (69.6)	56 (68.3)	139 (70.2)	0.951 (0.542–1.667)	0.860
Intrauterine growth restriction, No (%)	35 (12.5)	9 (10.9)	26 (13.1)	0.817 (0.365–1.831)	0.624
Pregnancy-induced hypertension, No. (%)	68 (24.3)	20 (24.4)	48 (24.2)	1.018 (0.558–1.856)	0.954
Gestational diabetes, No. (%)	27 (9.6)	6 (7.3)	21 (10.6)	0.672 (0.261–1.732)	0.408
Small for gestational age at birth, No. (%)	59 (21.1)	16 (19.5)	43 (21.7)	0.901 (0.473–1.715)	0.751
Twins, No. (%)	80 (28.6)	28 (34.1)	52 (26.3)	1.456 (0.835–2.537)	0.184
pH on cord blood	7.3 (7.2 to 7.3)	7.2 (7.2 to 7.3)	7.3 (7.2 to 7.3)	-	0.784
CRIB II score	6 (5 to 8)	9 (8 to 10)	5 (4 to 6)	-	<0.001
Mechanical Ventilation, No. (%)	226 (80.7)	78 (95.1)	148 (74.7)	6.588 (2.295–18.914)	<0.001
Invasive mechanical ventilation, No. (%)	89 (31.8)	46 (56.1)	43 (21.7)	4.606 (2.653–7.997)	<0.001
Non-invasive mechanical ventilation, No. (%)	220 (78.6)	74 (90.2)	146 (73.7)	3.295 (1.488–7.297)	0.002
Patent Ductus Arteriosus, No. (%)	73 (26.1)	36 (43.9)	37 (18.7)	3.405 (1.938–5.985)	<0.001

^a^ Intramuscular steroid cycle in two doses of 12 mg over a 24-h period; CRIB (clinical risk index for babies). # Cohort A vs. Cohort B. Data were expressed as mean (95% CI), when not specified.

**Table 2:** Morbidity of the entire study population.

	Total *n* = 280	Cohort A (HG) *n* = 82	Cohort B (Control) *n* = 198	OR (95% CI)	*p* Value #
Necrotizing enterocolitis	13 (4.6)	5 (6.1)	8 (4.0)	1.542 (0.489–4.862)	0.322
Intraventricular hemorrhage all stage	19 (6.8)	12 (14.6)	7 (3.6)	4.653 (1.761–12.294)	0.001
Intraventricular hemorrhage stage > II	11 (3.9)	8 (9.8)	3 (1.5)	7.027 (1.815–27.204)	0.003
Periventricular leukomalacia	7 (2.5)	3 (3.7)	4 (2.0)	1.842 (0.403–8.418)	0.336
Sepsis all diagnosis	26 (9.3)	14 (17.1)	12 (6.1)	3.191 (1.406–7.242)	0.004
Sepsis proven by positive culture	23 (8.2)	12 (14.6)	11 (5.6)	2.914 (1.230–6.908)	0.012
Retinopathy of prematurity all stage	52 (18.6)	26 (31.7)	26 (13.1)	3.071 (1.650–5.719)	<0.001
Retinopathy of prematurity stage ≥ II	40 (14.3)	21 (25.6)	19 (9.6)	3.243 (1.635–6.435)	<0.001
Bronchopulmonary dysplasia	18 (6.4)	12 (14.8)	6 (3.0)	5.536 (2.001–15.321)	<0.001
Overall morbidity	68 (24.3)	35 (42.7)	33 (16.7)	3.723 (2.094–6.620)	<0.001

# Cohort A vs. Cohort B. Data were expressed as No. (%).

**Table 3:** Neurodevelopmental outcome at 24 months of life of the study population.

	Overall		In TPN in the First Week of Life	
	Cohort A (HG) *n* = 32	Cohort B (Control) *n* = 76	Cohort A (HG) *n* = 25	Cohort B (Control) *n* = 39
**Cognitive scale**				
Scaled score	6.8 (6.0 to 7.7) *	7.9 (7.5 to 8.5)	6.6 (5.6 to 7.6)	7.8 (6.9 to 8.7)
Composite score	84.2 (80.1 to 88.3) *	89.9 (87.3 to 92.5)	83.0 (77.8 to 88.1)	88.9 (84.5 to 93.4)
**Language scale**				
Receptive Language	6.1 (5.4 to 6.8) *	7.1 (6.7 to 7.5)	5.9 (5.0 to 6.7) *	7.0 (6.3 to 7.7)
Expressive Language	6.3 (5.6 to 6.9)	7.0 (6.5 to 7.4)	5.8 (5.1 to 6.6) *	6.9 (6.2 to 7.6)
Total Scaled score	12.4 (11.1 to 13.7) *	14.1 (13.3 to 14.9)	11.7 (10.2 to 13.2) *	13.9 (12.6 to 15.2)
Total Composite score	78.0 (74.2 to 81.9) *	82.9 (80.6 to 85.3)	76.0 (71.5 to 80.6) *	82.5 (78.7 to 86.3)
**Motor scale**				
Fine Motor	8.9 (7.9 to 9.9)	10.2 (9.7 to 10.6)	8.2 (7.1 to 9.4) *	9.9 (9.2 to 10.7)
Gross Motor	7.0 (6.3 to 7.7) ***	8.3 (7.9 to 8.6)	6.6 (5.8 to 7.4) ***	8.1 (7.7 to 8.5)
Total Scaled score	15.9 (14.4 to 17.5) **	18.4 (17.8 to 19.1)	14.8 (13.2 to 16.5) **	18.1 (17.0 to 19.1)
Total Composite score	87.9 (83.3 to 92.6) **	95.4 (93.4 to 97.4)	84.5 (79.5 to 89.5) **	94.3 (91.1 to 97.4)

TPN (total parenteral nutrition); * vs. Cohort B *p*-value < 0.05; ** vs. Cohort B *p*-value < 0.01; *** vs. Cohort B *p*-value < 0.001. Data were expressed as mean (95% CI).

## Data Availability

Data are available upon reasonable request. All data relevant to the study are included in the article. Access to raw data would be provided upon request.

## Supplementary Materials

The following are available online at https://www.mdpi.com/article/10.3390/nu13061930/s1, Table S1: Baseline clinical characteristics of the newborns in total parenteral nutrition in the first week of life; Table S2: Morbidity of the newborns in total parenteral nutrition in the first week of life; Figure S1: Nutritional intake of the first week of life.
