# Supplementary material for: Neonatal Hyperglycemia Related to Parenteral Nutrition Affects Long-Term Neurodevelopment in Preterm Newborn: A Prospective Cohort Study
Source: Nutrients. 2021 Jun 4;13(6):1930. doi: 10.3390/nu13061930 (PMC8227040; doi:10.3390/nu13061930)
Supplement: Supplementary file 1 [file nutrients-13-01930-s001.zip › Figure supplementary.pdf]

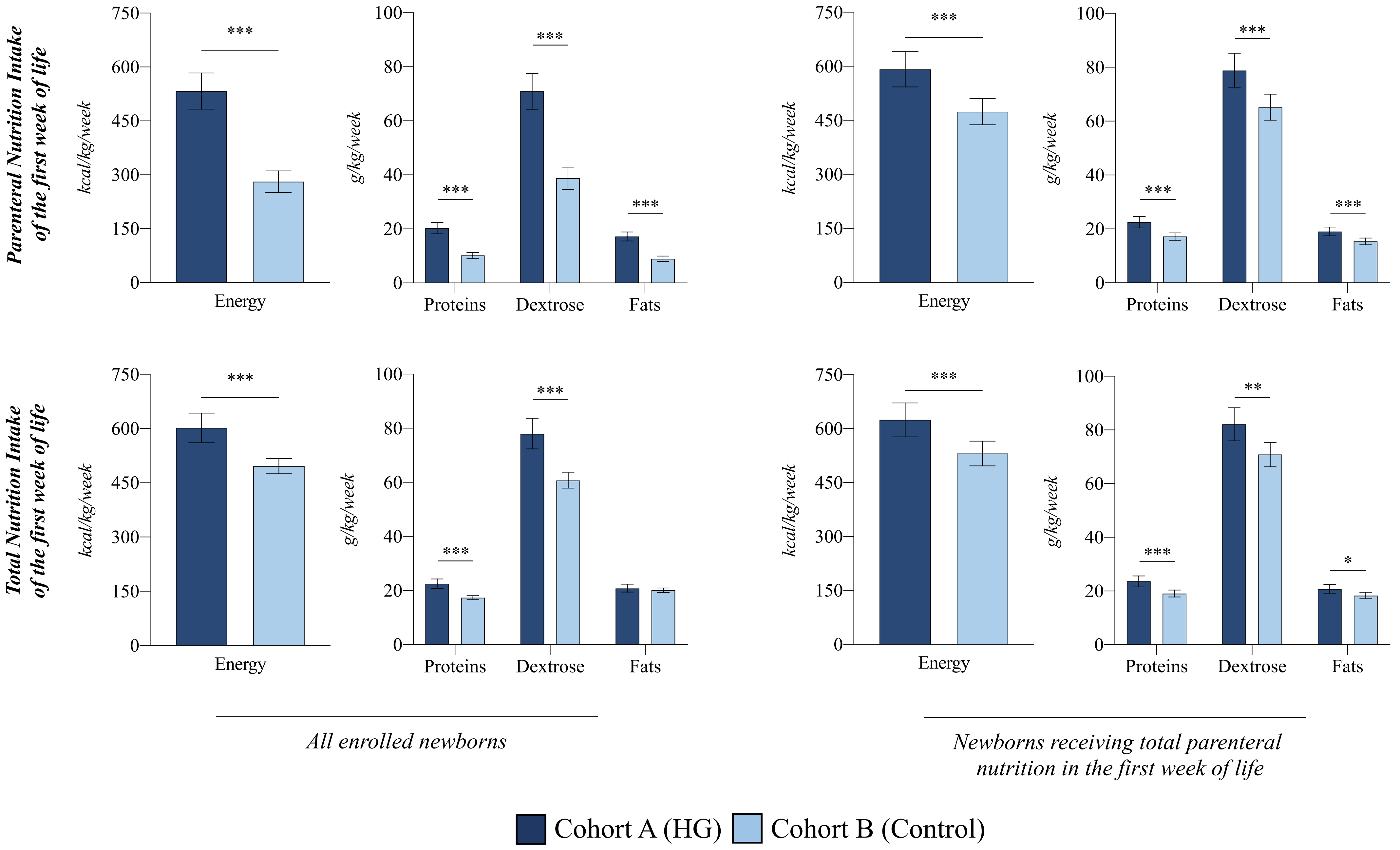

Parenteral Nutrition Intake of the first week of life
Total Nutrition Intake of the first week of life
kcal/kg/week
g/kg/week
750
600
450
300
150
0
100
80
60
40
20
***
**
*
Energy
Proteins
Dextrose
Fats
All enrolled newborns
Newborns receiving total parenteral nutrition in the first week of life
Cohort A (HG)
Cohort B (Control)
