# Supplementary material for: Neonatal Hyperglycemia Related to Parenteral Nutrition Affects Long-Term Neurodevelopment in Preterm Newborn: A Prospective Cohort Study
Source: Nutrients. 2021 Jun 4;13(6):1930. doi: 10.3390/nu13061930 (PMC8227040; doi:10.3390/nu13061930)
Supplement: Supplementary file 1 [file nutrients-13-01930-s001.zip › Tables Supplementary.pdf]

Table S1. Baseline clinical characteristics of the newborns in total parenteral nutrition in the first week of life.

	Total *n = 147*	Cohort A *n = 68*	Cohort B *n = 79*	OR (95% CI)	p value ¶
**Maternal age,** *years old*	34 (33 to 35)	34 (33 to 36)	35 (33 to 36)	-	0.460
**Maternal age ≥ 35 years old,** No. (%)	61 (41.4)	22 (32.4)	39 (49.4)	0.443 (0.222-0.887)	**0.021**
**Gestational age,** weeks	28 (27 to 29)	28 (27 to 28)	29 (29 to 30)	-	**< 0.001**
**Gestational age ≤ 29 weeks,** No. (%)	98 (66.7)	53 (77.9)	45 (57.0)	0.375 (0.181-0.774)	**0.007**
**Birth weight,** g	1075 (1022 to 1128)	957 (878 to1036)	1176 (1111 to 1241)	-	**< 0.001**
**Birth weight ≤ 1000 grams,** No. (%)	64 (43.5)	42 (61.8)	22 (27.8)	4.185 (2.092-8.375)	**< 0.001**
**Male sex,** No. (%)	83 (56.5)	38 (55.9)	45 (57.0)	0.957 (0.498-1.840)	0.895
**Cesarean section,** No. (%)	128 (87.1)	57 (83.8)	71 (89.9)	0.584 (0.220-1.548)	0.276
**Antenatal corticosteroids** [a]**,** No. (%)	96 (65.3)	46 (67.6)	50 (63.3)	1.270 (0.637-2.532)	0.496
**Intrauterine growth restriction,** No (%)	19 (12.9)	9 (13.2)	10 (12.7)	1.071 (0.408-2.813)	0.890
**Pregnancy-induced hypertension,** No. (%)	36 (24.5)	18 (26.5)	18 (22.8)	1.245 (0.586-2.645)	0.569
**Gestational diabetes,** No. (%)	9 (6.1)	3 (4.4)	6 (7.6)	0.570 (0.137-2.374)	0.336
**Small for gestational age at birth,** No. (%)	34 (23.1)	15 (22.1)	19 (24.1)	0.929 (0.429-2.012)	0.851
**Twins,** No. (%)	40 (27.2)	21 (30.9)	19 (24.1)	1.411 (0.681-2.924)	0.353
**pH on cord blood**	7.3 (7.2 to 7.3)	7.2 (7.2 to7.3)	7.2 (7.2 to7.3)	-	0.579
**CRIB II score**	8 (7 to 9)	9 (8 to 10)	7 (6 to 8)	-	**< 0.001**
**Mechanical Ventilation,** No. (%)	137 (93.2)	67 (98.5)	70 (88.6)	8.614 (1.062-69.853)	**0.016**
**Invasive mechanical ventilation,** No. (%)	71 (48.3)	41 (60.3)	30 (38.0)	2.480 (1.275-4.824)	**0.007**
**Non-invasive mechanical ventilation,** No. (%)	132 (89.8)	63 (92.6)	69 (87.3)	1.826 (0.592-5.633)	0.289
**Patent Ductus Arteriosus,** No. (%)	59 (40.1)	35 (51.5)	24 (30.4)	2.431 (1.237-4.775)	**0.009**

Figure Legend. (a) Intramuscular steroid cycle in two doses of 12 mg over a 24-hour period; CRIB (Clinical risk index for babies). ¶ Cohort A vs Cohort B. Data were expressed as mean (95% CI), when not specified.

Table S2. Morbidity of the newborns in total parenteral nutrition in the first week of life.

	Total *n = 147*	Cohort A *n = 68*	Cohort B *n = 79*	OR (95% CI)	*p* value ¶
**Necrotizing enterocolitis**	3 (2.0)	3 (4.4)	0 (0)	0.451 (0.377-0.540)	0.097
**Intraventricular hemorrhage all stage**	18 (12.2)	11 (16.2)	7 (8.9)	1.985 (0.723-5.446)	0.177
**Intraventricular hemorrhage stage > II**	11 (7.5)	8 (11.8)	3 (3.8)	3.378 (0.859-13.285)	0.067
**Periventricular leukomalacia**	4 (2.7)	2 (2.9)	2 (2.5)	1.167 (0.160-8.512)	0.632
**Sepsis all diagnosis**	19 (12.9)	12 (17.6)	7 (8.9)	2.204 (0.815-5.964)	0.113
**Sepsis proven by positive culture**	17 (11.6)	10 (14.7)	7 (8.9)	1.773 (0.636-4.947)	0.269
**Retinopathy of prematurity all stage**	44 (29.9)	25 (36.8)	19 (24.1)	1.836 (0.899-3.748)	0.093
**Retinopathy of prematurity stage ≥ II**	34 (23.1)	20 (29.4)	14 (17.7)	1.935 (0.888-4.213)	0.094
**Bronchopulmonary dysplasia**	16 (10.9)	11 (16.2)	5 (6.3)	2.868 (0.942-8.728)	0.055
**Overall morbidity**	50 (34.0)	32 (47.1)	18 (22.8)	3.012 (1.482-6.123)	**0.002**

Notes. ¶ Cohort A vs Cohort B. Data were expressed as No. (%).
